# A combined clinical and biomarker approach to predict diuretic response in acute heart failure

**DOI:** 10.1007/s00392-015-0896-2

**Published:** 2015-08-18

**Authors:** Jozine M. ter Maaten, Mattia A. E. Valente, Marco Metra, Noemi Bruno, Christopher M. O’Connor, Piotr Ponikowski, John R. Teerlink, Gad Cotter, Beth Davison, John G. Cleland, Michael M. Givertz, Daniel M. Bloomfield, Howard C. Dittrich, Dirk J. van Veldhuisen, Hans L. Hillege, Kevin Damman, Adriaan A. Voors

**Affiliations:** Department of Cardiology, University Medical Center Groningen, University of Groningen, Hanzeplein 1, 9713 GZ Groningen, The Netherlands; University of Brescia, Brescia, Italy; Department of Cardiovascular Sciences, “Sapienza” University of Rome, Rome, Italy; Duke University Medical Center, Durham, NC USA; Clinical Military Hospital, Medical University, Wroclaw, Poland; University of California at San Francisco and San Francisco Veterans Affairs Medical Center, San Francisco, CA USA; Momentum Research, Durham, NC USA; University of Hull, Kingston upon Hull, UK; Brigham and Women’s Hospital, Boston, MA USA; Merck Research Laboratories, Rahway, NJ USA; University of Iowa Carver College of Medicine Cardiovascular Research Center, Iowa City, IA USA; University Medical Center Groningen, Department of Epidemiology, University of Groningen, Groningen, The Netherlands

**Keywords:** Diuretic response, Heart failure, Biomarkers, Prediction

## Abstract

**Background:**

Poor diuretic response in acute heart failure is related to poor clinical outcome. The underlying mechanisms and pathophysiology behind diuretic resistance are incompletely understood. We evaluated a combined approach using clinical characteristics and biomarkers to predict diuretic response in acute heart failure (AHF).

**Methods and results:**

We investigated explanatory and predictive models for diuretic response—weight loss at day 4 per 40 mg of furosemide—in 974 patients with AHF included in the PROTECT trial. Biomarkers, addressing multiple pathophysiological pathways, were determined at baseline and after 24 h. An explanatory baseline biomarker model of a poor diuretic response included low potassium, chloride, hemoglobin, myeloperoxidase, and high blood urea nitrogen, albumin, triglycerides, ST2 and neutrophil gelatinase-associated lipocalin (*r*^2^ = 0.086). Diuretic response after 24 h (early diuretic response) was a strong predictor of diuretic response (*β* = 0.467, *P* < 0.001; *r*^*2*^ = 0.523). Addition of diuretic response after 24 h to biomarkers and clinical characteristics significantly improved the predictive model (*r*^*2*^ = 0.586, *P* < 0.001).

**Conclusions:**

Biomarkers indicate that diuretic unresponsiveness is associated with an atherosclerotic profile with abnormal renal function and electrolytes. However, predicting diuretic response is difficult and biomarkers have limited additive value. Patients at risk of poor diuretic response can be identified by measuring early diuretic response after 24 h.

**Electronic supplementary material:**

The online version of this article (doi:10.1007/s00392-015-0896-2) contains supplementary material, which is available to authorized users.

## Introduction

Treatment of acute heart failure (AHF) is primarily aimed at decongestion using diuretics. Suboptimal response to diuretics, or diuretic resistance, may reflect disease severity and is associated with impaired renal function and poor clinical outcome [1]. Recently, a definition for diuretic response was introduced, combining weight loss and diuretic dose, thus creating a quantitative indexed measure of diuretic response [2, 3]. Patients with a poor diuretic response had a significantly higher risk of post-discharge death or heart failure rehospitalization. Identification of patients with poor diuretic response or resistance early after hospital admission might lead to adaptation of treatment, potentially resulting in earlier relief of dyspnea, shorter length of hospital stay and—hypothetically—a reduced risk of hospital readmission early after discharge. Second, the pathophysiology behind the individual variation in diuretic response is not well understood. Valente et al. previously published the association of a poor diuretic response with more advanced heart failure, renal impairment, diabetes, and atherosclerotic disease [2]. Biomarkers might help in the early prediction of diuretic response during hospital admission, and may provide additional insights in the mechanisms underlying diuretic unresponsiveness. We, therefore, aimed to establish an explanatory and predictive model for diuretic response in acute heart failure using clinical characteristics and biomarkers from different pathophysiological domains.

## Methods

### Study design and procedures

The study population and design has previously been described in detail [4, 5]. In short, 2033 patients hospitalized for acute heart failure with mild to moderate renal dysfunction participated in the placebo-controlled randomized study of the selective A1 adenosine receptor antagonist rolofylline for patients hospitalized with acute decompensated heart failure and volume overload to assess treatment effect on congestion and renal function (PROTECT). The PROTECT study was a large, multicenter, phase III randomized clinical trial with neutral results. The trial was approved by the local ethics committee at each participating center. All patients provided written informed consent.

At baseline and after 24 h, signs and symptoms of heart failure were assessed, as well as standard laboratory assessments. We selected 26 biomarkers based on their known association with outcome or severity of heart failure, renal function and atherosclerosis. Endothelial cell-selective adhesion molecule (ESAM), growth differentiation factor 15 (GDF-15), lymphotoxin beta receptor (LTβR), mesothelin, osteopontin, mid regional pro-adrenomedullin (MR-proADM), receptor for advanced glycation endproducts (RAGE), soluble ST2, syndecan-1, and tumor necrosis factor alpha receptor 1 (TNF-R1a), were measured using sandwich enzyme-linked immunosorbent assays (ELISAs) on a Luminex^®^ platform by Alere Inc., San Diego, Ca, USA. By use of competitive ELISAS on a Luminex^®^ platform Angiogenin was measured. Finally, galectin-3, myeloperoxidase (MPO), and neutrophil gelatinase-associated lipocalin (NGAL) were measured using sandwich ELISAs on a microtiter plate. Two additional biomarkers, brain natriuretic peptide (BNP), and Kidney Injury molecule (KIM-1), were measured by single molecule counting technology by Erenna^®^ Immunoassay System on a microtiter plate by Singulex Inc., Alameda, CA, USA.

### Definition of diuretic response and study population

Diuretic response was defined as weight change on day 4 per 40 mg of furosemide (or equivalent doses) administered from baseline to day 3 as described earlier [2]. Early diuretic response is defined as weight change after 24 h per 40 mg of furosemide (or equivalent doses). Data on fluid intake, or urine output was not available. Of the 2033 included patients, 1288 patients had complete biomarker data available at baseline. A total of 1113 patients had additional complete biomarker data after 24 h. Of these, 974 patients also had data of diuretic response available. This study, therefore, included a total study population of 974 patients. The selected subpopulation did not significantly differ from the excluded patients (supplementary Table 1).

### Statistical analysis

The analyses were performed in the intention to treat population. Continuous variables are presented as mean ± standard deviation or median with (interquartile range) when appropriate. Categorical values are presented as frequencies and percentages. Differences between groups were tested for significance with ANOVA (normal distribution) and Kruskal–Wallis (skewed distribution). A linear trend was statistically tested over quintiles of diuretic response, after checking for non-linear trends.

Uni- and multivariable linear regression analysis was performed with transformed values when necessary. Transformations were checked using multifractional polynomials. As a good diuretic response implicates lower values, standardized beta’s need to be interpreted inverted, where a negative standardized beta means higher values are associated with a good diuretic response. Explanatory models were created based on statistical significance, whereas predictive models were selected on best fit [6]. Multivariable explanatory regression models, including all univariable variables with a *P* value <0.10, were constructed via backward elimination and validated using bootstrap re-sampling with 1000 replicates. The models were tested for collinearity and checked by plotting residuals. Finally, an internal bootstrap with 1000 replicates of the selected models was performed, testing stability of these models. Spearman’s rank correlation coefficient (*r*) was used to assess the relation between predicted diuretic response and diuretic response after 4 days.

A two tailed *P* value <0.05 was considered statistically significant. Analyses were performed using R: a Language and Environment for Statistical Computing, version 3.0.2. (R Foundation for Statistical Computing, Vienna, Austria).

## Results

Biomarkers at baseline per quintile of diuretic response are presented in Table 1. Renal biomarkers, such as serum creatinine, blood urea nitrogen (BUN), and plasma NGAL, and atherosclerotic biomarkers (ESAM and LTβR) showed significant trend over quintiles of diuretic response. Potassium, chloride, and sodium were significantly lower in patients with a poor response, whereas albumin and uric acid were significantly higher.

**Table 1:** Baseline biomarkers per quintile of diuretic response

Diuretic response (kg/40 mg furosemide):	−1.28 (−1.79 to 1.00)	−0.67 (−0.77 to 0.57)	−0.36 (−0.42 to 0.33)	−0.18 (−0.23 to 0.14)	0.00 (−0.04 to 0.20)	*P* trend
*N*	193	198	193	195	195	
Albumin (g/dL)	3.8 (3.6 to 4.1)	3.9 (3.6 to 4.1)	3.9 (3.6 to 4.2)	3.9 (3.7 to 4.2)	3.9 (3.6 to 4.2)	0.038
Angiogenin (ng/ml)	1896.9 (1300 to 2897.3)	2041.5 (1341.6 to 2975.5)	1984.8 (1392.5 to 2827.3)	1913.1 (1249.1 to 2757.7)	1806.2 (1273.4 to 2835.4)	0.599
Blood urea nitrogen (mg/dl)	27 (21 to 33)	28 (21 to 38)	28 (22 to 42)	35 (27 to 48)	33 (24 to 45)	<0.001
BNP (pg/ml)	419 (238.4 to 771.6)	451.7 (249.7 to 738.6)	476.9 (260.5 to 752.8)	467.5 (258.7 to 830.5)	463.4 (256 to 808.3)	0.338
Chloride (mEq/L)	103 (100 to 105)	102 (100 to 105)	102 (99 to 104)	100 (97 to 103.5)	100 (97 to 103)	<0.001
Total cholesterol (mg/dl)	150 (122 to 173)	149 (119 to 181)	144 (118 to 174)	137 (115.5 to 167)	139 (111 to 172)	0.022
Creatinine (mg/dl)	1.3 (1.1 to 1.6)	1.3 (1.1 to 1.7)	1.4 (1.1 to 1.7)	1.5 (1.2 to 2)	1.4 (1.2 to 1.8)	<0.001
ESAM (ng/ml)	59.6 (55.2 to 66.8)	60.8 (56.2 to 68.4)	60.9 (56.4 to 67.2)	63.2 (58.1 to 70.8)	63.1 (57.2 to 71.3)	0.001
Galectin-3 (ng/ml)	31.8 (24.9 to 41.6)	32.8 (25.1 to 45.4)	35.4 (27.4 to 47.4)	39 (30 to 49.5)	37 (27.4 to 48.5)	<0.001
GDF-15 (ng/ml)	4.1 (2.8 to 6.3)	3.8 (2.8 to 6)	4.1 (3 to 6.3)	5.1 (3.5 to 6.3)	5.1 (3.6 to 6.3)	<0.001
Hemoglobin (g/dL)	12.9 (11.6 to 14.2)	12.9 (11.3 to 14.2)	12.5 (11.2 to 13.6)	12.3 (11.1 to 13.6)	12.4 (11.1 to 13.8)	0.003
KIM 1 (pg/ml)	271.1 (174.4 to 380.5)	247.6 (179.8 to 439.3)	297.5 (164.9 to 518.1)	317.8 (205 to 540.7)	310.5 (190.3 to 481.7)	0.652
LTBR (ng/ml)	0.4 (0.3 to 0.5)	0.4 (0.3 to 0.5)	0.4 (0.3 to 0.6)	0.5 (0.3 to 0.6)	0.5 (0.3 to 0.7)	<0.001
Mesothelin (ng/ml)	85.2 (72.4 to 97.9)	84.3 (71.8 to 96.9)	84.8 (73.8 to 97.6)	89.3 (77.8 to 102)	90.2 (77.7 to 103.6)	0.006
MR-proADM (ng/ml)	2.6 (1.6 to 3.9)	2.7 (1.5 to 4.5)	2.8 (1.5 to 4)	3 (1.5 to 5.6)	3 (1.5 to 5.5)	0.009
Myeloperoxidase (ng/ml)	37.2 (19.6 to 71)	36.9 (19.2 to 75.2)	30.4 (18.6 to 59)	29.1 (17.3 to 55.5)	29.3 (16.8 to 54.5)	0.005
NGAL (ng/ml)	70.9 (49.1 to 111.8)	72.9 (52.2 to 108.4)	81.2 (57.1 to 116.4)	97.2 (56.7 to 160.3)	91.2 (54.3 to 155.1)	<0.001
Osteopontin (ng/ml)	107.3 (74.9 to 162.1)	108.9 (76.6 to 160.7)	108.4 (74.3 to 155.4)	119.6 (83.8 to 157.4)	123.8 (88.5 to 174.8)	0.113
Potassium (mmol/L)	4.3 (3.9 to 4.6)	4.3 (3.9 to 4.7)	4.3 (3.9 to 4.7)	4.2 (3.8 to 4.7)	4.1 (3.8 to 4.5)	0.001
RAGE (ng/ml)	5 (3.6 to 6.5)	5 (3.5 to 6.6)	5.3 (3.8 to 6.9)	5.1 (4 to 7.8)	5 (3.9 to 6.8)	0.034
Sodium (mmol/L)	141 (138 to 143)	140 (138 to 143)	140 (138 to 142)	139 (137 to 142)	139 (136.5 to 142)	<0.001
ST-2 (ng/ml)	2.7 (0.9 to 6.3)	3.1 (0.9 to 7.1)	2.6 (0.9 to 7.5)	4.7 (1.8 to 9.3)	4.5 (1.4 to 9.3)	0.039
Syndecan-1 (ng/ml)	7.9 (6.8 to 9.4)	8.2 (7 to 9.6)	8.2 (6.9 to 9.7)	8.9 (7.5 to 11.2)	8.8 (7.4 to 10.5)	<0.001
TNF-R1a (ng/ml)	2.7 (2.1 to 3.7)	2.9 (2.2 to 4.2)	2.9 (2.1 to 4.2)	3.7 (2.6 to 5.2)	3.5 (2.6 to 5.3)	<0.001
Triglycerides (mmol/L)	78 (59 to 109)	92 (68.2 to 117)	90 (67 to 130)	89 (67 to 131)	82 (63 to 127)	0.011
Uric acid (mg/dl)	8.5 (7.2 to 10.1)	8.2 (6.9 to 10.1)	8.8 (7.4 to 10.7)	9 (7.5 to 10.8)	9.4 (7.6 to 11.2)	<0.001

*BNP* brain natriuretic peptide, *ESAM* endothelial cell-selective adhesion molecule, *GDF-15* growth differentiation factor 15, *KIM-1* kidney injury molecule 1, *LTβR* lymphotoxin beta receptor, *MR-proADM* mid regional pro-adrenomedullin, *NGAL* neutrophil gelatinase-associated lipocalin, RAGE receptor for advanced glycation end products, *TNF-R1a* tumor necrosis factor alpha receptor 1

A similar pattern was observed for biomarker levels after 24 h (supplementary Table 2). Baseline characteristics of this population per quintile of diuretic response are presented in supplementary Table 3. In brief, poor responders had a lower blood pressure, more frequent diabetes and ischemic heart disease. An explanatory multimarker biomarker model included albumin, BUN, chloride, hemoglobin, MPO, NGAL, potassium, ST2, and triglycerides, but yielded only marginal explanatory value of diuretic response (*r*^2^ = 0.086). Higher chloride, hemoglobin, MPO and potassium levels were associated with a good diuretic response (Table 2). In addition, a good diuretic response was also associated with lower levels of albumin, BUN, NGAL, ST2, and triglycerides.

**Table 2:** Explanatory biomarker baseline model

Variable	Beta coefficient	95 % CI	*T* value	*P* value
Albumin (per SD)	0.072	0.03 to 0.12	3.045	0.002
Log blood urea nitrogen (per SD)	0.076	0.02 to 0.13	2.797	0.005
Chloride (per SD)	−0.066	−0.11 to −0.02	−2.772	0.006
Hemoglobin (per SD)	−0.058	−0.11 to −0.01	−2.308	0.021
Myeloperoxidase (per SD)	−0.060	−0.11 to −0.01	−2.490	0.013
NGAL (per SD)	0.064	0.01 to 0.12	2.307	0.021
Potassium (per SD)	−0.103	−0.15 to −0.06	−4.333	<0.001
ST2 (per SD)	0.054	0.01 to 0.10	2.213	0.027
Triglycerides (per SD)	0.060	0.02 to 0.10	2.814	0.005

*r* ^2^ = 0.086

*NGAL* neutrophil gelatinase-associated lipocalin

In addition, an explanatory model for biomarkers after 24 h included BUN, hemoglobin, MPO, sodium, ST2, and triglycerides (*r*^2^ = 0.082). Again, higher hemoglobin, MPO, and sodium levels, and lower levels of BUN, ST2, and triglycerides were associated with a good diuretic response (supplementary Table 4). Finally, an explanatory clinical baseline model (Table 3) showed that good diuretic response was associated with higher systolic blood pressure, higher weight and JVP, less frequent history of Diabetes Mellitus, PCI, COPD, beta blocker, and metolazone use, and more spironolactone use and randomized rolofylline treatment (*r*^2^ = 0.134).

**Table 3:** Explanatory clinical baseline model

Variable	Beta coefficient	95 % CI	*T* value	*P* value
Weight (per SD)	−0.089	−0.13 to −0.04	−3.781	<0.001
Systolic blood pressure (per SD)	−0.082	−0.13 to −0.04	−3.648	<0.001
Rolofylline treatment	−0.138	−0.23 to −0.05	−2.999	0.003
Jugular venous pressure	−0.107	−0.19 to −0.02	−2.386	0.017
Diabetes mellitus	0.159	0.07 to 0.25	3.490	0.001
PCI	0.181	0.08 to 0.28	3.558	<0.001
COPD	0.127	0.02 to 0.23	2.294	0.022
Beta blocker	0.207	0.10 to 0.31	3.963	<0.001
Spironolactone	−0.131	−0.22 to −0.04	−2.869	0.004
Metolazone	0.317	0.14 to 0.50	3.432	0.001

*r* ^2^ = 0.134

*PCI* percutaneous coronary intervention, *COPD* chronic obstructive pulmonary disease

**Fig. 1:**
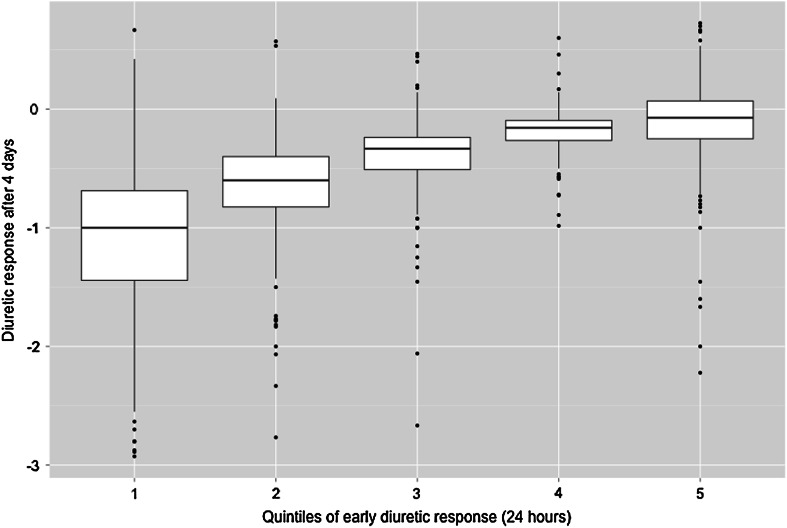
Diuretic response after 4 days per quintile of diuretic response after 24 h

**Fig. 2:**
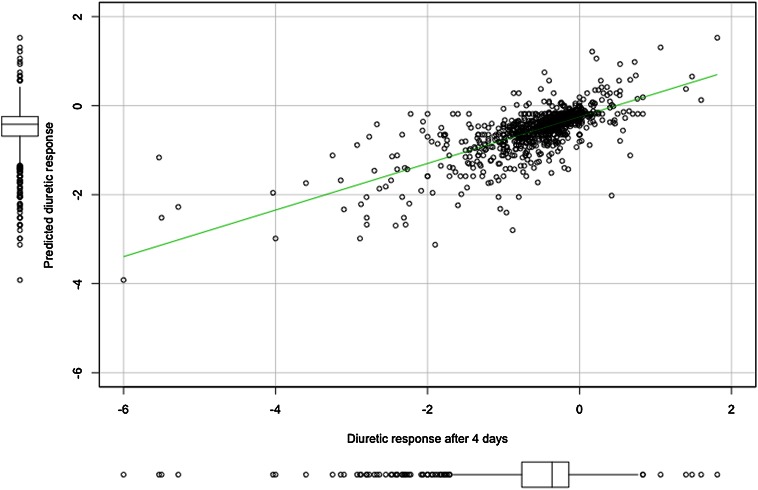
Scatter plot of predicted response and calculated diuretic response after 4 days

Exploration of a model that contained variables 24 h after randomization identified *early* diuretic response (after 24 h) as a strong predictor of good diuretic response (univariable *β* = 0.467, *P* < 0.001; *r*^*2*^ = 0.523) at 4 days. In Fig. 1 median diuretic response and interquartile ranges after 4 days are plotted per quintile of *early* diuretic response from, respectively, good (quintile 1) to poor response (quintile 5). This figure shows that a poor *early* response had reasonable consistency with diuretic response values after 4 days. Prediction of diuretic response at day 4 based on early diuretic response alone showed a strong correlation (*r* = 0.723, *P* < *0.001*). The scatter plot of predicted response (based on the early response after 24 h) and measured diuretic response on day 4 is shown in Fig. 2. Out of 974 patients, 98 patients (10.1 %) had a good *early* diuretic response after 24 h (>median) and a poor response on day 4 (≤median). Clinical characteristics and biomarkers revealed no important differences between this group and other groups based on response after 24 h and on day 4.

Based on these findings, we explored a 24-h predictive model for diuretic response (Table 4). In addition to early diuretic response (after 24 h), BUN at baseline, change in BUN (24 h—baseline), systolic blood pressure, weight at baseline and randomized rolofylline treatment, as well as a history of COPD, Diabetes Mellitus, PCI, and JVP were included in the model. The use of spironolactone, and beta blockers, baseline potassium, triglycerides, chloride, and MPO, and ST2, and hemoglobin after 24 h further provided a significant improvement of fit (*r*^*2*^ = 0.586, *P* < 0.001). Internal bootstrapping gave similar results.

**Table 4:** 24 hour diuretic response prediction model

Variable	Beta coefficient	95 % CI	*T* value	*P* value
Early diuretic response (after 24 h) (per SD)	0.479	0.45 to 0.51	28.052	<0.001
Systolic blood pressure (per SD)	−0.068	−0.10 to −0.04	−4.208	<0.001
Change in blood urea nitrogen (24 h—baseline) (per SD)	0.055	0.02 to 0.09	3.403	0.001
Potassium at baseline (per SD)	−0.053	−0.08 to −0.02	−3.207	0.001
COPD	0.111	0.03 to 0.19	2.869	0.004
Triglycerides at baseline (per SD)	0.038	0.01 to 0.07	2.646	0.008
Diabetes mellitus	0.079	0.01 to 0.14	2.397	0.017
Beta blocker	0.088	0.02 to 0.16	2.393	0.017
Weight (per SD)	−0.037	−0.07 to −0.01	−2.279	0.023
Spironolactone	−0.073	−0.14 to −0.01	−2.262	0.024
Jugular venous pressure	−0.072	−0.13 to −0.01	−2.31	0.021
PCI	0.074	0.00 to 0.14	2.059	0.040
Log blood urea nitrogen at baseline (per SD)	0.033	0.00 to 0.07	1.920	0.055
Rolofylline treatment	−0.060	−0.12 to 0.00	−1.883	0.060
ST2 after 24 h (per SD)	0.029	0.00 to 0.06	1.778	0.076
Hemoglobin after 24 h (per SD)	−0.026	−0.06 to 0.01	−1.586	0.113
Chloride at baseline (per SD)	−0.021	−0.05 to 0.01	−1.274	0.203
Myeloperoxidase at baseline (per SD)	−0.019	−0.05 to 0.01	−1.152	0.250

*r* ^2^ = 0.586

*COPD* chronic obstructive pulmonary disease, *PCI* percutaneous coronary intervention

## Discussion

Poor response to diuretics in patients admitted with acute heart failure is a clinical problem associated with high morbidity and mortality. Using a panel of clinical and biochemical markers, we aimed to determine a non-response profile for two reasons. First, it is of clinical relevance to predict patients at risk, to initiate early alternative therapies [7]. Second, a distinct clinical or biochemical profile might provide more information about a pathophysiological mechanism behind diuretic unresponsiveness.

### Identification of patients at risk of poor diuretic response

Clinical characteristics and biomarkers fall short in predicting diuretic response. However, assessment of early diuretic response after 24 h allows the clinician to identify patients at risk of diuretic resistance shortly after hospital admission. Although it is probably not surprising that diuretic response at day 4 is predicted by early diuretic response after only 24 h, it provides important clinical application. This readily applicable metric can be used daily in all patients hospitalized for acute heart failure. Implementation of this metric in clinical practice will identify both patients with favorable diuretic response and patients with diuretic resistance early on during hospitalization. Once either of these patients are identified, treatment strategies may be adapted. Alternative and sometimes more aggressive strategies can be explored in patients with significant unresponsiveness with a great risk of adverse outcome, as these patients are more likely to benefit from alternative therapies. Several strategies can be considered. One of these is combination diuretic therapy—addition of a thiazide diuretic or a mineralocorticoid receptor antagonist at natriuretic doses—can help overcome diuretic resistance and improve natriuresis [8–11]. Other alternatives are for instance, adding dopamine or switching to ultrafiltration [7]. Although the larger all-comer trials were neutral for these therapies, both of these approaches have been insufficiently investigated in truly unresponsive, diuretic resistant patients. Our analyses also showed that randomized allocation to rolofylline, an adenosine A-1 antagonist, was associated with a good diuretic response. This might suggest that, in specific subpopulations, rolofylline may help overcome poor diuretic response. Whether this will also improve outcome is unknown. Future studies should aim to investigate the effects of alternative strategies on relieve of dyspnea and clinical outcome in patients admitted for acute heart failure.

### Predictors of diuretic response

Many clinical variables and biomarkers are related to diuretic response. Our analyses show that a poor diuretic response is strongly associated with renal and atherosclerotic biomarkers, like creatinine, NGAL, ESAM and LTβR. The clinical characteristics, previously described by Valente et al. provided the same results in this smaller subset of patients from the PROTECT cohort [2]. Atherosclerotic characteristics as well as higher levels of novel atherosclerotic biomarkers like ESAM and LTβR were associated with a poor response. In the Dallas Heart study ESAM was associated with subclinical atherosclerosis; while LTβR also associated with multiple signs of atherosclerosis in this study, confirming that a link between these markers and poor diuretic response could be pointing towards a phenotype with atherosclerotic properties [12, 13].

Interestingly, renal biomarkers, such as creatinine, BUN and NGAL, were more abnormal over increasing quintiles of diuretic response and were significant predictors of diuretic response. Renal tubular function is of key importance for diuretic efficacy [14]. The finding that higher levels of plasma NGAL were associated with a poor diuretic response supports this. However, as plasma KIM-1 was not significantly associated with diuretic response; the question remains whether circulating NGAL and KIM-1 levels both reflect tubular function [15]. A higher creatinine and BUN level was associated with a poor response to diuretic treatment. Ferreira et al. previously identified plasma urea as a predictor of slower diuretic response [16]. In our study, an increase in BUN is also predictive of poor diuretic response, suggesting not only baseline values but also worsening of renal function is of influence on diuretic response. Both, renin–angiotensin–aldosterone system and sympathetic nervous system activation cause a flow-dependent passive resorption of urea in the distal tubule, caused by increased sodium and water resorption in the proximal tubule [17, 18]. This consequently results in diminished distal flow and increased reabsorption. Elevated BUN levels, therefore, indicate a kidney working actively to retain water and sodium. This could be one of the reasons for the far greater increase in BUN compared with serum creatinine with poorer diuretic response. In addition, loop diuretics need to be actively secreted by the organic anion transporter in the proximal tubule to arrive at their site of action at the luminal side of the tubule [19, 20]. Organic anions, like uric acid, competitively bind this receptor, thus causing diminished diuretic availability [21, 22].

Several electrolytes, like potassium, sodium and chloride showed associations with diuretic response. Interestingly, higher chloride levels were associated with a better diuretic response. High chloride levels have been shown to reduce renin release and increase blood pressure [23]. In addition, loop diuretics inhibit the reabsorption of chloride in the loop of Henle. A higher chloride level may, therefore, slightly suppress the renin-angiotensin-aldosterone system, hence possibly increasing renal perfusion and sodium reabsorption, and provide a less depleted chloride level during diuretic treatment. Similarly, a low potassium was associated with a poor diuretic response, likely due to its co-transporter function.

Finally, MPO, ST2 and NGAL were the only ‘novel’ biomarkers in the multivariable models of diuretic response. Interestingly, both ST2 and MPO are thought to be associated with a pro-inflammatory state. Higher MPO levels are associated with more advanced heart failure and adverse outcome in chronic heart failure patients [24]. In patients with acute coronary syndrome, pre-admission treatment with statins, beta blockers or ACE inhibitors reduced MPO levels [25]. In this study we paradoxically found an association between low MPO levels and poor diuretic response. Higher ST2 levels have been found in chronic kidney disease patients and correlated with disease severity [26]. Addition of ST2 to BNP in acute heart failure patients has been shown to improve prognostic accuracy [27].

### Limitations

This study is a retrospective analysis of a randomized clinical trial. Unfortunately, not all patients had complete biomarker data available at baseline and after 24 h, creating a selected subpopulation used for these analyses. In addition, these analyses are data driven and causality cannot be proven. The results of this study need to be validated in a different population. Research assays to MR-proADM, galectin-3, and ST2 were developed by Alere, and have not been standardized to the commercialized assays used in research or in clinical use. The extent to which each Alere assay correlates with the commercial assay is not fully characterized. Information on fluid intake, urine output or net fluid balance was not collected in the PROTECT database. We were unfortunately not able to compare diuretic response based on weight loss to other metrics, for instance based on urine excretion or net fluid loss.

## Conclusions

Biomarkers indicate that poor diuretic response is associated with a profile of atherosclerosis, glomerular and tubular renal dysfunction and abnormal electrolytes. These markers were of limited clinical use to predict diuretic response at hospital admission for acute heart failure. Patients at risk of diuretic resistance can be identified by measuring diuretic response after 24 h.

## Electronic supplementary material

Supplementary material 1 (DOCX 45 kb)

## Abbreviations

AHF: Acute heart failure
BNP: Brain natriuretic peptide
BUN: Blood urea nitrogen
ESAM: Endothelial cell-selective adhesion molecule
GDF-15: Growth differentiation factor 15
KIM-1: Kidney injury molecule 1
LTβR: Lymphotoxin beta receptor
MPO: Myeloperoxidase
MR-proADM: Mid regional pro-adrenomedullin
NGAL: Neutrophil gelatinase-associated lipocalin
PROTECT: Placebo-controlled randomized study of the selective A1 adenosine receptor antagonist rolofylline for patients hospitalized with acute decompensated heart failure and volume overload to assess treatment effect on congestion and renal function
RAGE: Receptor for advanced glycation endproducts
TNF-R1a: Tumor necrosis factor receptor 1
