# Supplementary material for: A combined clinical and biomarker approach to predict diuretic response in acute heart failure
Source: Clin Res Cardiol. 2015 Aug 18;105:145–53. doi: 10.1007/s00392-015-0896-2 (PMC4735256; doi:10.1007/s00392-015-0896-2)
Supplement: Supplementary file 1 — Supplementary material 1 (DOCX 45 kb) [file 392_2015_896_MOESM1_ESM.docx]

**Supplementary material**

**A combined clinical and biomarker approach to predict diuretic response in acute heart failure**

Clinical Research in Cardiology

Jozine M. ter Maaten, Mattia A.E. Valente, Marco Metra, Noemi Bruno, Christopher M. O’Connor, Piotr Ponikowski, John R. Teerlink, Gad Cotter, Beth Davison, John G. Cleland, Michael M. Givertz, Daniel M. Bloomfield^j^, Howard C. Dittrich, Dirk J. van Veldhuisen, Hans L. Hillege, Kevin Damman, Adriaan A. Voors

**Corresponding author:**

A.A. Voors, MD, PhD
Professor of Cardiology
University Medical Center Groningen

Hanzeplein 1, 9713 GZ Groningen, The Netherlands

Tel +31 (0)50 361 2355

[a.a.voors@umcg.nl](mailto:a.a.voors@umcg.nl)

*Supplementary table 1: Baseline characteristics of the included subpopulation and excluded subpopulation*

| **Variable** | **Excluded subpopulation** | **Included subpopulation** | **P-trend** |
| --- | --- | --- | --- |
| **N =** | 1059 | 974 |  |
| **Demographics** |  |  |  |
| **Sex (%(n) Male)** | 66.9 (709) | 67.2 (655) | 0.886 |
| **Age (years)** | 70±12 | 70.3±11.1 | 0.505 |
| **BMI (kg/m2)** | 29±6.3 | 28.7±6 | 0.410 |
| **LVEF (%(n))** | 32.5±13.4 | 32.2±12.8 | 0.769 |
| **HFPEF (%(n))** | 21.2 (106) | 18.3 (87) | 0.259 |
| **Systolic Blood Pressure (mmHg)** | 124.1±17.7 | 124.5±17.5 | 0.612 |
| **Diastolic Blood Pressure (mmHg)** | 73.4±12 | 74±11.7 | 0.258 |
| **Heart Rate (beats/min)** | 79.5±15.1 | 80.7±15.8 | 0.089 |
| **Rolofylline administration (%(n))** | 67.1 (711) | 66.2 (645) | 0.661 |
| **Clinical Profile** |  |  |  |
| **Atrial fibrillation on presentation** | 39.3 (170) | 43.6 (167) | 0.209 |
| **Orthopnea (%(n))** | 96 (994) | 96 (926) | 0.927 |
| **Rales (%(n))** | 58.2 (610) | 63.9 (622) | 0.008 |
| **Edema (%(n))** | 68.3 (718) | 67.5 (657) | 0.678 |
| **Jugular venous pressure (%(n))** | 39.2 (370) | 42.1 (372) | 0.202 |
| **Medical History** |  |  |  |
| **Hypertension (%(n))** | 79.2 (839) | 79.7 (776) | 0.804 |
| **Diabetes Mellitus (%(n))** | 44.7 (473) | 46.1 (449) | 0.529 |
| **Hypercholesterolemia (%(n))** | 54.2 (573) | 49.5 (482) | 0.035 |
| **Smoking (%(n))** | 22.6 (239) | 18.6 (180) | 0.026 |
| **Ischemic Heart Disease (%(n))** | 69.2 (732) | 70.5 (685) | 0.529 |
| **Myocardial Infarction (%(n))** | 49.2 (520) | 49.5 (481) | 0.913 |
| **PCI (%(n))** | 26 (273) | 25.8 (249) | 0.921 |
| **CABG (%(n))** | 20.8 (219) | 22.5 (217) | 0.363 |
| **Peripheral Vascular Disease (%(n))** | 10.9 (115) | 10.8 (105) | 0.962 |
| **Atrial Fibrillation (%(n))** | 56 (589) | 53.1 (514) | 0.185 |
| **NYHA Class (%(n))** |  |  |  |
| **I-II** | 18.2 (179) | 17.5 (165) | ref. |
| **III** | 51.5 (507) | 50.5 (475) | 0.897 |
| **IV** | 30.3 (298) | 32.0 (301) | 0.500 |
| **ICD therapy (%(n))** | 16.1 (170) | 15.9 (155) | 0.925 |
| **CRT therapy (%(n))** | 10.8 (114) | 9.7 (94) | 0.408 |
| **Stroke (%(n))** | 9.1 (96) | 8.9 (87) | 0.917 |
| **COPD (%(n))** | 20.3 (214) | 19.3 (188) | 0.594 |
| **Prior Medication Use** |  |  |  |
| **ACE inhibitors or ARB (%(n))** | 76.1 (802) | 75.2 (732) | 0.624 |
| **Beta blockers (%(n))** | 76.3 (804) | 76.2 (742) | 0.958 |
| **Mineralocorticoid Receptor Antagonists (%(n))** | 40.2 (423) | 47.7 (465) | 0.001 |
| **Calcium Antagonists (%(n))** | 13.9 (146) | 13.2 (129) | 0.677 |
| **Nitrates (%(n))** | 25.6 (269) | 26.4 (257) | 0.676 |
| **Digoxin (%(n))** | 25.9 (273) | 30.4 (296) | 0.025 |
| **Laboratory Values** |  |  |  |
| **Creatinine (mg/dL)** | 1.4 [1.1-1.8] | 1.4 [1.1-1.8] | 0.734 |
| **Glomerular Filtration Rate (ml/min/1.73m2)** | 48.8 [36.5-63.5] | 48.9 [37.2-63.5] | 0.789 |
| **Blood Urea Nitrogen (mg/dL)** | 29 [22-41] | 29 [22-41] | 0.638 |
| **Sodium (mmol/L)** | 140 [137-142] | 140 [137-142] | 0.044 |
| **Potassium (mmol/L)** | 4.2 [3.9-4.6] | 4.2 [3.9-4.6] | 0.768 |
| **Hemoglobin (g/dL)** | 12.8±2.1 | 12.6±1.9 | 0.087 |
| **Anemia (%(n))** | 41.5 (329) | 43.3 (422) | 0.450 |
| **Total Cholesterol (mmol/L)** | 146.1±44.8 | 148.2±43.7 | 0.289 |
| **Triglycerides (mmol/L)** | 101.3±52 | 101.7±58.6 | 0.849 |
| **NT-proBNP (pg/mL)** | 3000 [3000-3702] | 3000 [3000-3879.5] | 0.525 |

*Abbreviations: BMI: body mass index, LVEF: left ventricular ejection fraction, HFpEF: heart failure with preserved ejection fraction, PCI: percutaneous coronary intervention, CABG: coronary artery bypass surgery, NYHA: New York heart association, ICD: implantable cardiac defibrillator, CRT: cardiac resynchronization therapy, COPD: chronic obstructive pulmonary disease, angiotensin converting enzyme.*

*Supplementary table 2: Biomarker values after 24 hours per quintile of diuretic response*

| Diuretic response (kg/40 mg furosemide) | -1.28 [-1.79--1.00] | -0.67 [-0.77--0.57] | -0.36 [-0.42--0.33] | -0.18 [-0.23--0.14] | 0.00 [-0.04-0.20] | *P-trend* |
| --- | --- | --- | --- | --- | --- | --- |
| N = | 193 | 198 | 193 | 195 | 195 |  |
| Albumin (g/dL) | 3.8 [3.5-4] | 3.8 [3.4-4.1] | 3.8 [3.6-4.2] | 3.8 [3.6-4] | 3.8 [3.5-4.1] | 0.164 |
| Angiogenin (ng/ml) | 2103.3 [1371.9-3129.3] | 1992.2 [1295.8-3032.8] | 2235.6 [1439.7-2945] | 1935.2 [1310.1-2693.3] | 1859.6 [1227.4-2783.9] | 0.062 |
| Blood Urea Nitrogen (mg/dl) | 25 [20-32] | 27 [20-37] | 29 [22-39] | 36 [26.5-48.5] | 34 [25-46] | <0.001 |
| Chloride (mEq/l) | 101 [99-104] | 102 [98-104] | 101 [98-104] | 99 [96.5-102] | 100 [97-103] | <0.001 |
| Total cholesterol (mg/dl) | 156 [127-187] | 153.5 [124-185.8] | 147 [123-181] | 144 [119.5-173] | 140 [114-175.5] | <0.001 |
| Creatinine (mg/dl) | 1.3 [1.1-1.6] | 1.3 [1-1.7] | 1.4 [1.1-1.7] | 1.5 [1.2-2] | 1.5 [1.2-1.8] | <0.001 |
| ESAM (ng/ml) | 59.8 [54.4-66.8] | 61 [55.5-69.5] | 59.5 [53.4-68.9] | 63.5 [57.5-70.4] | 64.3 [57.6-72] | <0.001 |
| Galectin-3 (ng/ml) | 31.8 [25-42.1] | 33.8 [25.8-43.6] | 36.3 [27.8-48.6] | 39.9 [30.7-50.5] | 36.7 [27.8-48.1] | 0.001 |
| GDF-15 (ng/ml) | 3.8 [2.6-5.8] | 3.7 [2.6-6.3] | 3.8 [2.7-5.5] | 5 [3.5-6.3] | 5 [3.2-6.3] | <0.001 |
| Hemoglobin (g/dL) | 13.2 [12-14.6] | 12.9 [11.3-14.3] | 12.7 [11.4-13.9] | 12.2 [10.9-13.6] | 12.3 [10.9-13.9] | <0.001 |
| LTBR (ng/ml) | 0.4 [0.3-0.5] | 0.4 [0.3-0.5] | 0.4 [0.2-0.6] | 0.4 [0.3-0.7] | 0.4 [0.3-0.7] | <0.001 |
| Mesothelin (ng/ml) | 82.9 [69.6-93.5] | 83.3 [73-100.4] | 82.2 [68.9-99] | 88.3 [75.8-104.2] | 87.9 [74.8-101.7] | 0.003 |
| Myeloperoxidase (ng/ml) | 39.8 [21.2-78.9] | 35.6 [20.1-73.9] | 35.6 [22.1-62.1] | 31.5 [17.6-58.1] | 28.9 [17.5-58.4] | 0.007 |
| NGAL (ng/ml) | 68.8 [47.5-118] | 80.2 [51.1-124.9] | 78.9 [58.4-122.6] | 97.1 [56.4-151] | 90.4 [59.8-145.1] | 0.002 |
| Osteopontin (ng/ml) | 101.4 [67.3-145.5] | 101.4 [71.3-155] | 107.5 [75.7-160.2] | 124.2 [85.9-185.2] | 122.2 [93.2-171.8] | <0.001 |
| Potassium (mmol/L) | 4.1 [3.8-4.5] | 4.2 [3.9-4.6] | 4.2 [3.8-4.6] | 4.2 [3.8-4.5] | 4.1 [3.8-4.5] | 0.926 |
| proADM (ng/ml) | 2.4 [1.5-3.6] | 2.4 [1.3-3.9] | 2.2 [1.2-3.9] | 2.8 [1.5-4.9] | 3 [1.4-5.4] | <0.001 |
| RAGE (ng/ml) | 4.1 [3.1-5.8] | 4.6 [3.3-6.1] | 4.4 [3.2-6.2] | 5 [3.7-6.6] | 4.8 [3.4-6.6] | 0.010 |
| Sodium (mmol/L) | 141 [139-143] | 140 [138-143] | 140 [138-142] | 139 [136.5-141] | 139 [136.5-141] | <0.001 |
| ST-2 (ng/ml) | 1.1 [0.9-3.8] | 1.8 [0.9-4.7] | 1.6 [0.9-4.5] | 2.8 [0.9-7.6] | 2.6 [0.9-7.8] | <0.001 |
| Syndecan-1 (ng/ml) | 8.1 [6.6-9.6] | 8.2 [6.9-9.6] | 8.1 [6.8-9.8] | 8.9 [7.6-10.6] | 8.5 [7-10.5] | <0.001 |
| TNF-R1a (ng/ml) | 2.7 [2-3.8] | 2.9 [2.1-4.2] | 3 [2-4.4] | 3.5 [2.4-5.4] | 3.6 [2.5-5] | <0.001 |
| Triglycerides (mmol/L) | 88 [60-126] | 103 [73-138.5] | 100 [75-137] | 100 [76-155] | 91 [65-133] | 0.194 |
| Uric acid (mg/dl) | 8.8 [7.5-10.3] | 8.8 [7.1-10.5] | 9.1 [7.8-11.2] | 9.3 [7.8-11.2] | 9.6 [7.8-11.3] | <0.001 |

**Triglycerides significant quadratic trend*

*Abbreviations: ESAM: endothelial cell-selective adhesion molecule, GDF-15: growth differentiation factor 15, LTβR: lymphotoxin beta receptor, NGAL: neutrophil gelatinase-associated lipocalin, RAGE: receptor for advanced glycation end products, TNF-R1a: tumor necrosis factor alpha receptor 1.*

*Supplementary table 3: Baseline characteristics per quintile of diuretic response*

| Diuretic response (kg/40 mg furosemide) | -1.28 [-1.79--1.00] | -0.67 [-0.77--0.57] | -0.36 [-0.42--0.33] | -0.18 [-0.23--0.14] | 0.00 [-0.04-0.20] | *P-trend* |
| --- | --- | --- | --- | --- | --- | --- |
| N = | 193 | 198 | 193 | 195 | 195 |  |
| Demographics |  |  |  |  |  |  |
| Sex (%(n) Male) | 67.9 (131) | 67.2 (133) | 62.2 (120) | 75.9 (148) | 63.1 (123) | 0.936 |
| Age (years) | 70.6±11 | 69.9±11 | 71±10.6 | 69.8±10.9 | 70.4±12 | 0.825 |
| BMI (kg/m2) | 28.9±6.1 | 28.8±6.2 | 28.9±5.8 | 28.3±5.2 | 28.6±6.5 | 0.425 |
| LVEF (%(n)) | 34.6±11.9 | 33±12.3 | 31.7±12.5 | 28.4±12.6 | 33.2±13.9 | 0.065 |
| HFPEF (%(n)) | 24 (23) | 19.4 (18) | 14.6 (14) | 10.9 (10) | 22.4 (22) | 0.379 |
| Systolic Blood Pressure (mmHg) | 128.1±15.8 | 125.3±17.1 | 125.6±17.5 | 121.4±17.7 | 122±18.6 | <0.001 |
| Diastolic Blood Pressure (mmHg) | 77±11.6 | 74.9±11 | 74.9±11.1 | 71.6±11.1 | 71.8±12.7 | <0.001 |
| Heart Rate (beats/min) | 82.6±17.8 | 80.9±15.7 | 81.1±15.3 | 79.3±14.5 | 79.5±15.2 | 0.032 |
| Rolofylline administration (%(n)) | 75.6 (146) | 63.1 (125) | 68.4 (132) | 64.1 (125) | 60 (117) | 0.005 |
| Clinical Profile |  |  |  |  |  |  |
| Atrial fibrillation on presentation (%(n)) | 54.3 (44) | 47.6 (39) | 41.4 (29) | 34.2 (25) | 39 (30) | 0.013 |
| Orthopnea (%(n)) | 93.2 (177) | 98 (192) | 94.8 (182) | 96.4 (187) | 97.4 (188) | 0.127 |
| Rales (%(n)) | 64.8 (125) | 61.9 (122) | 63.7 (123) | 66.2 (129) | 63.1 (123) | 0.935 |
| Edema (%(n)) | 70.5 (136) | 73.2 (145) | 67.9 (131) | 63.6 (124) | 62.1 (121) | 0.012 |
| Jugular venous pressure (%(n)) | 48.5 (83) | 44.5 (77) | 38.4 (68) | 39.6 (72) | 40 (72) | 0.064 |
| Medical History |  |  |  |  |  |  |
| Hypertension (%(n)) | 79.3 (153) | 79.8 (158) | 81.3 (157) | 77.9 (152) | 80 (156) | 0.964 |
| Diabetes Mellitus (%(n)) | 33.7 (65) | 39.9 (79) | 52.3 (101) | 54.4 (106) | 50.3 (98) | <0.001 |
| Hypercholesterolemia (%(n)) | 40.4 (78) | 41.4 (82) | 51.3 (99) | 57.4 (112) | 56.9 (111) | <0.001 |
| Smoking (%(n)) | 14.1 (27) | 12.7 (25) | 19.4 (37) | 23.6 (46) | 23.1 (45) | 0.001 |
| Ischemic Heart Disease (%(n)) | 62.7 (121) | 70.1 (138) | 71.9 (138) | 74.4 (145) | 73.3 (143) | 0.014 |
| Myocardial Infarction (%(n)) | 48.2 (93) | 43.1 (85) | 43.8 (84) | 56.4 (110) | 55.9 (109) | 0.011 |
| PCI (%(n)) | 11.6 (22) | 21.1 (41) | 23.6 (45) | 37.1 (72) | 35.4 (69) | <0.001 |
| CABG (%(n)) | 13.2 (25) | 17.9 (35) | 23.7 (45) | 29.7 (58) | 27.7 (54) | <0.001 |
| Peripheral Vascular Disease (%(n)) | 10.4 (20) | 6.6 (13) | 10.5 (20) | 13.3 (26) | 13.3 (26) | 0.071 |
| Atrial Fibrillation (%(n)) | 60.7 (116) | 58.9 (116) | 45.1 (87) | 51.8 (101) | 49 (94) | 0.007 |
| NYHA Class (%(n)) |  |  |  |  |  | 0.697 |
| I-II | 15.5 (28) | 21.5 (42) | 19.3 (36) | 16.2 (31) | 15.0 (28) |  |
| III | 49.7 (90) | 44.1 (86) | 46.5 (87) | 55.0(105) | 57.2 (107) |  |
| IV | 34.8 (63) | 34.4 (67) | 34.2 (64) | 28.8 (55) | 26.8 (52) |  |
| ICD therapy (%(n)) | 7.3 (14) | 12.1 (24) | 16.6 (32) | 21 (41) | 22.6 (44) | <0.001 |
| CRT therapy (%(n)) | 6.2 (12) | 7.1 (14) | 9.9 (19) | 14.4 (28) | 10.8 (21) | 0.014 |
| Stroke (%(n)) | 8.3 (16) | 8.1 (16) | 8.8 (17) | 9.2 (18) | 10.3 (20) | 0.431 |
| COPD (%(n)) | 14.5 (28) | 16.7 (33) | 18.1 (35) | 20.1 (39) | 27.2 (53) | 0.001 |
| Prior Medication Use |  |  |  |  |  |  |
| ACE inhibitors or ARB (%(n)) | 74.6 (144) | 75.8 (150) | 77.2 (149) | 71.3 (139) | 76.9 (150) | 0.990 |
| Beta blockers (%(n)) | 64.2 (124) | 71.7 (142) | 79.3 (153) | 82.6 (161) | 83.1 (162) | <0.001 |
| Mineralocorticoid Receptor Antagonists (%(n)) | 51.8 (100) | 45.5 (90) | 45.6 (88) | 48.2 (94) | 47.7 (93) | 0.634 |
| Calcium Antagonists (%(n)) | 11.9 (23) | 15.2 (30) | 14.5 (28) | 14.4 (28) | 10.3 (20) | 0.586 |
| Nitrates (%(n)) | 22.3 (43) | 25.8 (51) | 24.9 (48) | 27.7 (54) | 31.3 (61) | 0.046 |
| Digoxin (%(n)) | 31.1 (60) | 32.8 (65) | 27.5 (53) | 28.2 (55) | 32.3 (63) | 0.832 |

*Abbreviations: BMI: body mass index, LVEF: left ventricular ejection fraction, HFpEF: heart failure with preserved ejection fraction, PCI: percutaneous coronary intervention, CABG: coronary artery bypass surgery, NYHA: New York heart association, ICD: implantable cardiac defibrillator, CRT: cardiac resynchronization therapy, COPD: chronic obstructive pulmonary disease, angiotensin converting enzyme.*

*Supplementary table 4: Explanatory biomarker model after 24 hours*

| Variable | Beta Coeff | 95% CI | T value | *P-value* |
| --- | --- | --- | --- | --- |
| Log Blood Urea Nitrogen (per SD) | 0.108 | (0.06-0.16) | 4.494 | <0.001 |
| Hemoglobin (per SD) | -0.066 | (-0.11--0.02) | -2.780 | 0.006 |
| Myeloperoxidase (per SD) | -0.079 | (-0.12--0.03) | -3.477 | 0.001 |
| Sodium (per SD) | -0.064 | (-0.11--0.02) | -2.677 | 0.008 |
| ST2 (per SD) | 0.072 | (0.02-0.12) | 2.918 | 0.004 |
| Triglycerides (per SD) | 0.063 | (0.02-0.11) | 2.930 | 0.003 |

*r^2^=0.082*
